# The Importance of General Therapeutics in the Treatment of Ocular Diseases

**Published:** 1907-08-10

**Authors:** A. Maitland Ramsay

**Affiliations:** Surgeon Ophthalmic Institution and Consulting Ophthalmic Surgeon, Royal Infirmary, Glasgow


### Hospital Clinics.

### THE IMPORTANCE OF GENERAL THERAPEUTICS IN THE TREATMENT OF OCULAR DISEASES.

By A. MAITLAND RAMSAY, M.D., F.F.P.S.G., Surgeon Ophthalmic Institution and Consulting Ophthalmic Surgeon, Royal Infirmary, Glasgow.

For most men it is easier to put trust in authority than to exercise independent judgment, and so instead of thinking for themselves they accept a clearly expressed opinion as a scientific fact, or even mistake a mere impression for a real opinion.

While it must never be forgotten that treatment is an art, and is none the less valuable because it is empirical, yet the whole trend of modern progress is to appeal less and less to authority. We endeavour in all things to go to Nature direct, and by patient research to find out her secrets. Investigation of this kind demands much time and labour, and the life of man is short, and his mental and physical capacity for work limited. If any real advance is to be made, the extent of the subject and the briefness of existence make subdivision of labour necessary, and specialism is thus the natural outcome of increase of knowledge. It is an inevitable result of evolution, and, as an ideal, ought to be symbolic of the highest form of efficiency.

A specialist in the true sense of the word is one who has conscientiously studied the body as a whole, and the highest and best type is he whose anatomical and physiological knowledge is most perfect, and whose early training has fitted him to take broad and comprehensive views of pathology. For all efficient higher training there must be a proper foundation, and consequently the specialist in medicine must combine in himself the diagnostic skill of the physician and the dexterity of the surgeon, and must add to these the special knowledge that gives him power and action reaching still farther along his own particular line. If, therefore, specialism is to render the highest service to the world, it must of necessity be based on an efficient system of general instruction; and consequently it is incumbent upon every specialist to strive, by reading, by frequent visits to the wards of a general hospital, and by conversation with colleagues, to keep in touch with the progress of pathology along at least its most important lines of advance.

The fashion of the present day is to speak of a pure specialist, but although he may devise new means of local treatment, or improve methods of operative technique, there is a risk of his doing so without regard to the broad principles which underlie all rational therapeutics. I would not for a moment under-estimate the value of local treatment; to do so would be to run counter to all the knowledge of the process of inflammation which we have gained through the study of bacteriology. What I desire to emphasise is the need for assigning to local treatment no more than its proper value. It is just possible that the great amount of consideration given in recent years to the study of micro-organisms has tended to divert attention unduly from the soil on which the bacteria grow. For example, in surgical work one must not only endeavour to render the field of operation sterile, but must also devote the utmost care and attention to what is of equal importance—the giving the patient himself increased power of resistance. Good food, fresh air, rest, every means of strengthening digestion, are all essential. Should the sufferer be a poor man, who has been unfit for work for several months, and through poverty unable to procure a sufficient amount of food, he will require special care, since his tissues will be in the condition most favourable for the action of even the slightest infective taint, and also for the rapid spread of suppuration. While, therefore, every attention must be paid to strict antiseptic precautions at the time of the operation, equal care should be bestowed on the state of the patient himself, especially if he be poor and habitually ill-nourished. Were the health of these broken-down subjects better looked after prior to cataract and other operations there would be fewer failures from suppuration.

The results of infection depend not only upon the germ, but also on the soil on which the micro-organism is sown. For example, if an ulcer form on the cornea of a healthy man the probabilities are that it can easily be cured by local treatment, whereas if the ulcer occur in an unhealthy person micro-organisms are not confined to the seat of injury, but are found along with their toxins both in the tissues and in the blood; consequently, if a speedy recovery is to be brought about, there must be general as well as local treatment. In the one case it might be said that the cornea was injured, but in the other that the patient was diseased, and that the ulcer of the cornea is only a symptom of his unhealthy state. Success or failure will depend on a proper distinction between these two conditions.

All true advance in the treatment of diseases of the eye can be attained only by a careful study of the principles of general pathology. There can be no special study of morbid conditions in the eye, apart from post-mortem appearances incident to peculiarities of anatomical structure. Nor could we expect it to be otherwise when we remember how quickly sight is affected in all disordered states of the body, and how frequently actual pathological changes occur in the ocular tissues as consequences of general ailments. Happily, modern pathology is much more than a record of post-mortem appearances. It deals not only with structure, but also with function, and thereby takes cognisance of the origin and course, as well as of the results, of a morbid process. Its whole trend is to demonstrate, what has all along been known to the clinical observer, that a disease can cure itself. This all-sufficient power of Nature to bring a patient back from sickness to health is the very first lesson in rational therapeutics. It is recorded of Sydenham that when he was in doubt he consulted his own reputation and the patient's safety by doing nothing at all. Doubtless expectant treatment can be carried too far; but probably most will agree that many eyes are lost from injudicious meddling which might have been saved had they been left to Nature. The wonderful powers possessed by the natural restorative agencies of the living body were, and are still, spoken of as the "Constitution," or the "*Vis medicatrix naturæ*," but these words, although they encourage a physician to hold his hand, and in consequence oftentimes save a patient from the discomforts, and even the dangers, of what has been called active treatment, are nevertheless simply cloaks for ignorance. The new nomenclature which is arising in connection with the study of infection and immunity is, however, promising to provide us with a means of expressing ourselves intelligibly regarding the manner in which Nature works.

Undoubtedly prophylaxis is the highest form of therapeutics, and of late, both by the cultivation of sanitary science and by the practicable demonstration of the use of antitoxins in the treatment of diseases, great advances have been made in this direction. The study of the former, by bringing prominently forward the laws governing health, teaches not only individuals, but also whole communities, the value of an abundant supply of fresh air and of pure water, combined with a perfect system of drainage; while the latter has shown that the blood, when it is supplied with a quantity of a specially prepared serum, is reinforced by those very constituents of which it is most in need, and without which the recovery of the patient is impossible. The study of antitoxins is as yet in its infancy, and so far we possess specifics of this kind antagonistic to only a few diseases; but there is no reason to doubt that with the advance of science and the increase of knowledge serum treatment will come to bulk more and more largely in medical practice, until, perchance, we may at length be provided with a serum whose use at the right time will prevent that greatest of all ocular dangers—sympathetic ophthalmia.

The highest ideal of our profession is to discover and deal with all the causes of a disease, and these can be found only through a thorough knowledge of pathology. The more limited, therefore, the pathological outlook, the more deficient will be the general treatment, and so it follows that therapeutics to be rational must be founded on an accurate knowledge of the natural history of the ailment.

In my own experience there is nothing of more value in checking the progress of myopia in the young than a well-regulated course of Swedish gymnastics, and what is true of myopia is equally true of every form of ametropia. It is not enough, more especially when one is dealing with children, simply to adjust glasses suitable for the correction of the error of refraction. A quick estimate must at the same time be made of the patient's general strength, for it is most important to remember that, though the demand for work made upon the ciliary muscle may not be unreasonably great in itself, yet it may be altogether excessive in relation to the individual in whom it is made. While, therefore, the error of refraction must always be carefully corrected, so that the ciliary muscle may do its duty under the best possible conditions, every effort should also be made to develop and strengthen the whole muscular system by sufficiency of proper food, by drill, by exercise on the bicycle, or, preferably, on horseback, and by an active outdoor life.

In the eye, just as in any other part of the body, the limits of muscular capacity must be respected, and the amount of work to be done must be adjusted accordingly. Day after day at the Glasgow Ophthalmic Institution I see children sent from the poorest schools of the city by their teachers "to get spectacles." In nearly every case an error of refraction exists, but in many it is so slight that in all probability it would cause no trouble were the general health satisfactory. Most of these children are underfed, live in unsanitary houses, are the victims of unwholesome habits, and are in consequence physically unfit to bear the strain of continuous school work. Obviously, under these circumstances, it is wiser to prescribe rest for the eyes and treatment to improve the general health than simply to supply glasses, which by the relief they afford encourage the child to struggle on, only to have a more serious breakdown afterwards.

It follows, then, that I have no sympathy whatever with those who desire to separate optical from medico-surgical ophthalmology. The eye is not only in the body, but it is also of the body, and the proper treatment of its optical defects requires the physician's skill just as much as does the treatment of its diseases. On the other hand, it cannot be insisted on too often, or too strongly, that all these optical defects should be corrected early and thoroughly; for eye strain is accompanied by symptoms as numerous as they are varied; and its persistence is, in consequence, attended by grave dangers to the general health.

Whenever it is recognised that disease is due to the perversion of natural processes the path to its prevention or relief is at once made easier, for it is obvious that the very first indication for treatment is to maintain, or to improve, the nutrition of the body. The patient must be supplied with food of proper quality, and in sufficient quantity to satisfy his needs. With assimilation there is also always excretion. The corpuscles take up their food supply from the blood or from the lymph, absorb what they require, and reject the surplus. If the health of the cell is to be maintained at a proper standard, provision must be made for the complete elimination of these waste products; for if they be retained in the blood a state of general toxæmia will soon supervene, and this, if it persist for a considerable time, even to a slight extent, will, by lowering the state of the corpuscular health, seriously interfere with the resisting powers of the body as a whole. If, therefore, the nutritive value of the blood is to be maintained at the normal, it is imperative that all toxins be removed at the earliest possible moment, and to this end Nature has made provision in the bowel, the liver, the kidneys, the skin, and the lungs. The constant and free activity of these channels of excretion is essential to health, and if, by reason of illness, the function of one be temporarily interrupted, the others are always ready to undertake vicarious duty.

A very short clinical experience will, however, prove that the value of their respective functional activity varies in different morbid conditions. For example, in the early stages of diseases of the uveal tract a mercurial purge and alkaline saline draughts stimulate the liver, bowels, and kidneys, and are often attended by the most satisfactory results; in retro-bulbar neuritis relief is more quickly obtained by free diaphoresis; and in toxic amblyopia due to the abuse of alcohol and tobacco (the causes of the trouble having been removed, and digestive disturbances corrected by appropriate means) vision returns most speedily under the strengthening influence of a sea voyage, or of a sojourn in the dry, bracing air of the mountains—conditions in which the blood receives a plentiful supply of oxygen.

One of the most familiar examples of the evil result of improper feeding, accompanied by defective elimination, is found in phlyctenular conjunctivitis, and I therefore select the treatment of this disease as a practical illustration of what I have been saying. The sufferers are very numerous, and are for the most part children under ten years of age. Although some of them may present the outward appearance of good health, most of them are, owing to defective assimilation and excretion, either abnormally pale or unnaturally livid. They have but too obviously inherited the miserable legacy of constitutional weakness; and the inherent vulnerability of their tissues has been intensified by improper dieting and by overcrowding in unsanitary houses. The eye trouble very often follows an attack of measles or other of the exanthemata, and although under favourable circumstances it may speedily subside, it has a most characteristic tendency to recur, unless every cause be taken in hand from the very outset. If left alone, or simply treated by local remedies, the ophthalmia often proves to be the very first warning of the imminent danger of tubercular infection. This is especially likely to happen in those patients in whom photophobia is most distressing, yet without gross lesions of the conjunctiva or cornea.

It is by no means unusual for the conjunctivitis to alternate with suppuration of the middle ear, or with eczematous eruptions on the head or other parts of the body. Nutrition is seriously interfered with, and the coated tongue studded with large red papillæ shows the irritable condition of the mucous membrane of the whole intestinal tract. The urine is high-coloured, deposits urates, and often contains traces of sugar. The skin is pale and rough, the flesh hangs loosely on the bones, the hands and feet are often persistently cold, and the head perspires freely. After a time the system becomes infected by tubercle bacilli, and then pulmonary and intestinal troubles may supervene, and the joints, both large and small, are very frequently destroyed by tubercular inflammation of their synovial membrane followed by caries and necrosis of the bones. Severe ectropion—causing hideous disfigurement—wi[illegible] arise if the bone necrosis occur in the neighbourhood of the orbit; and indelible scars invariably result if there be suppuration of the lymphatic glands in the neck or elsewhere.

Unless the cornea has, through neglect, become ulcerated, local treatment consists simply in keeping the child's face and hands clean, and in applying sedatives to the eyes. The real treatment of the disease is constitutional. It is necessary always to bear in mind that the very essence of the ailment lies in the mal-assimilation of food, and that most, if not all, cases arise as a result of improper dieting. You may be told that the child pecks and trifles with its food, and eats very sparingly, and that may be quite true so far as the regular meals are concerned; but by careful inquiry you will usually discover that the small appetite at the proper times for eating arises from the patient having been liberally supplied between meals with bread and butter, sweets, fruit, or any other eatables that have chanced to be at hand. Many think that a good appetite is a sign of good digestion, though all the time the latter is so poor that in the overladen bowels toxic substances are constantly being manufactured, and these, after they are absorbed excite abnormal effects both in the eyes and in all the other tissues of the body. Instead, therefore, of eating too little, these children eat too much; and although they may appear ill-nourished, the mal-nutrition is due to mal-assimilation rather than to insufficiency of food. The first thing to be done, therefore, in treating a case of phlyctenular ophthalmia is to give particular directions regarding the diet. The child must not be allowed to eat whenever it cries for food, but ought to be fed at regular intervals. Nor must it get what is among the poor popularly called "the run of the house"; it should have meals specially prepared, and in quantity and quality of such a kind as not to overtax the powers of digestion. Sugar in every shape and form, fruits, pastries, potatoes, etc., must be rigidly excluded from the dietary, and it is usually advisable to confine the patient wholly to milk and water for the first forty-eight hours of the treatment, and then to add gradually meat-soups, eggs, fish, fowl, rice, etc., as the tongue becomes clean.

In addition to the careful supervision of diet, the free elimination of the toxins, which, as a result of mal-assimilation, have accumulated in the blood, must be promoted in every way. This is to be accomplished by stimulating the action of the kidneys, the bowels, and the skin—more especially the last. Hence the importance of the hot bath, which the child should have every night at bedtime, diaphoresis being further encouraged by the wearing of a warm flannel nightgown. It is usually advisable first of all to administer a dose of castor oil; but it should be borne in mind that many of these children do not bear purgatives well, as the intestinal mucous membrane is so irritable that troublesome diarrhœa is easily induced.

Of drugs, none is so useful as tartar emetic, which should be given, as Mackenzie advised, in slightly nauseating doses. Its chief virtue depends on its diaphoretic action, and its efficacy is greatly increased when it is administered with a laxative, *e.g.*, powdered rhubarb. If the tongue be brown, a few grains of grey powder may, with advantage, be added to the rhubarb and antimony combination. Under this treatment it is wonderful how soon the whole appearance of the patient changes. As the power of digestion becomes greater, the skin improves in colour, the hair loses its dry and brittle character, the hands and feet keep warmer, and the child, instead of lying with its face buried in a pillow, and fretting and crying when spoken to, is now quite good tempered, and runs about and amuses itself with its playthings. Any indiscretion in diet will, however, promptly bring on a relapse, for in these cases the conjunctiva reflects the conditions of the gastro-intestinal mucous membrane even more quickly than the tongue. After some forbidden food has been eaten, intolerance of light, blepharospasm, and increased lachrymation speedily show themselves, while the tongue may not become furred till some days after. This makes it imperative, therefore, that treatment be continued for at least a fortnight after the recovery seems complete. As soon as possible the child ought to be sent to the country, preferably to a high, dry, bracing locality; and it should live out of doors as much as the weather will permit. The sea-coast should be avoided, as the glare from the water is apt to prove irritating to the eyes, and to cause a relapse. In an ordinary case of phlyctenular ophthalmia no further treatment than that just indicated is required. If a case be not making satisfactory progress, careful inquiry should at once be made as to whether the prescribed diet and the nightly warm bath are being strictly attended to.

When the powers of digestion, respiration, and excretion become healthy, fewer restrictions as to diet are necessary, and a plan of treatment may then be entered upon to improve the general nutrition still further, and to promote the repair of any local lesions in the eyes themselves. Such restorative methods aim at supplying the blood with something specially needed at the moment, but if they were to be commenced prematurely they would do much harm by adding to the child's difficulty in assimilating food. In every attempt to assist recuperation the all-important indication is to stimulate appetite and to strengthen digestion by means of simple tonics, and on no account to prescribe iron, phosphates, cod-liver oil, or the hypophosphites if the tongue be either coated with fur or red and irritable.

The guiding principles applicable in the treatment of phlyctenular conjunctivitis apply with equal force to the general treatment of all eye diseases of constitutional origin. The first condition of success is to find out what is at the root of the trouble—be it syphilis, gout, rheumatism, or anything else—to prescribe appropriate remedies in such dose and state of combination as will suit the individual patient, and then to persevere steadily until the disease is overcome. The all-important point is to make a searching diagnosis early, so that from the first the treatment may be both local and constitutional. Neglect may bring about complications, and, of course, if the tissues be destroyed, no amount of anti-syphilitic, anti-rheumatic, or any other kind of medicine can replace them. In many instances, on the other hand, when, to begin with, the prospects of success are not hopeful, earnest perseverance will accomplish much, while intermittent and unsettled efforts will altogether fail.

What I have just said must be taken as applying to the line of treatment rather than to any particular drug, for it cannot be too strongly urged that, no matter how high the reputation a remedy may have, it at once does harm to the individual for whom it has been prescribed if it interfere with his powers of digestion and assimilation of food. It is a matter of common observation that the sight of those suffering from atrophy of the optic nerve, or from disease of the choroid or retina, deteriorates much and quickly whenever there is gastro-intestinal disturbance. He is, as a rule, the most successful practitioner who can at once vary the preparation and the combination of the drugs to suit the peculiarities of the patient, and at the same time never depart from the line of treatment he has laid down for himself. Such resourcefulness on the part of the doctor is of the greatest value to the patient, and I would add that in medicine, probably more than in any other profession, the personality of the practitioner counts for much.

Before concluding I must refer briefly to rest, in so far at least as it is an aid to nutrition. During tranquil sleep the restorative powers of Nature work undisturbed, and in calculating the risks of an operation Paget very wisely emphasised the fact that a patient's capacity for sleep was of the utmost importance. I do not recollect ever having had serious trouble with an operation case when the patient slept soundly for several hours immediately after being taken back to bed; and in order to induce post-operative slumber it is my usual practice, half-an-hour before the time fixed for the operation, to administer a draught containing the triple bromides. On the other hand, when a patient is wakeful and talkative, and keeps constantly tossing about in bed, it is almost impossible for the wound to heal by first intention. Unless means be quickly discovered to keep him quiet, it is usually wiser to remove the bandage and allow him to leave the recumbent position and to sit on a chair, when he may obtain the needful rest. One of the reasons why pain does so much harm after an operation is owing to the restlessness it induces; consequently, if I think there is much chance of a patient suffering, my practice is to give a small dose of morphia hypodermically. This, by ensuring several hours of continuous repose, gives Nature a healing chance of which she is never slow to avail herself to the uttermost.

It is not good practice, however, always to subdue pain, for it is, if rightly interpreted, oftener kindly than hurtful. As a rule it is a danger signal, warning both surgeon and patient of something wrong. For example, if pain recur some days after a serpiginous ulcer has been carefully cauterised, it is almost certain that reinfection has taken place, and the prompt application of local antiseptics may save much future trouble.

What is wanted in this very advanced age of medicine is not so much more learning, but the more exact application of what we have. We require to be made more conscious of the power of knowledge properly employed. Our experience teaches us facts, but it is only when, through a study of pathology, we get an explanation of the laws and principles underlying the facts, that we can act with a well-defined purpose, and can utilise our knowledge in the highest interest of humanity.

